# Which curve provides the best explanation of the growth in confirmed COVID-19 cases in Chile?^*^

**DOI:** 10.1590/1518-8345.4493.3346

**Published:** 2020-06-26

**Authors:** Víctor Díaz-Narváez, David San-Martín-Roldán, Aracelis Calzadilla-Núñez, Pablo San-Martín-Roldán, Alexander Parody-Muñoz, Gonzalo Robledo-Veloso

**Affiliations:** 1 Universidad Andres Bello, Facultad de Odontología, Santiago, Chile.; 2 Universidad de Valparaíso, Facultad de Medicina, Escuela de Obstetricia y Puericultura, Valparaíso, Chile.; 3 Universidad Bernardo OHiggins, Facultad de Salud, Santiago, Chile.; 4 Universidad Mayor, Facultad de Ciencias, Escuela de Nutrición y Dietética, Santiago, Chile.; 5 Universidad Metropolitana, Barranquilla, Colombia.; 6 Universidad de Chile, Facultad de Ciencias, Santiago, Chile.

**Keywords:** COVID-19, Coronavirus, 2019-nCoV, Coronavirus Infections, Pandemics, Epidemiology, COVID-19, Coronavirus, 2019-nCoV, Infecções por Coronavirus, Pandemias, Epidemiologia, COVID-19, Coronavirus, 2019-nCoV, Infecciones por Coronavirus, Pandemias, Epidemiología

## Abstract

**Objective:**

to explore the best type of curve or trend model that could explain the epidemiological behavior of the infection by COVID-19 and derive the possible causes that contribute to explain the corresponding model and the health implications that can be inferred.

**Method:**

data were collected from the COVID-19 reports of the Department of Epidemiology, Ministry of Health, Chile. Curve adjustment studies were developed with the data in four different models: quadratic, exponential, simple exponential smoothing, and double exponential smoothing. The significance level used was α≤0.05.

**Results:**

the curve that best fits the evolution of the accumulated confirmed cases of COVID-19 in Chile is the doubly-smoothed exponential curve.

**Conclusion:**

the number of infected patients will continue to increase. Chile needs to remain vigilant and adjust the strategies around the prevention and control measures. The behavior of the population plays a fundamental role. We suggest not relaxing restrictions and further improving epidemiological surveillance. Emergency preparations are needed and more resource elements need to be added to the current health support. This prediction is provisional and depends on keeping all intervening variables constant. Any alteration will modify the prediction.

## Abstract (pt)

**Objetivo:**

explorar o melhor tipo de curva ou modelo de tendência para explicar o comportamento epidemiológico do contágio por COVID-19 e derivar as possíveis causas que contribuem para explicar o modelo correspondente e as implicações em saúde que se podem inferir.

**Método:**

os dados foram coletados nos relatórios da COVID-19 do Departamento de Epidemiologia, Ministério da Saúde do Chile. Os dados foram analisados através do ajustamento de curvas em quatro modelos diferentes: quadrático, exponencial, suavização exponencial simples e suavização exponencial dupla. O nível significância adotado foi de α≤0.05.

**Resultados:**

a curva que mais se ajusta à evolução dos casos confirmados acumulados da COVID-19 no Chile é a curva com suavização exponencial dupla.

**Conclusão:**

o número de infectados continuará aumentando e o Chile deve permanecer vigilante e ajustar suas estratégias em torno de medidas de prevenção e controle. O comportamento da população tem um papel fundamental. Sugerimos não relaxar quanto às restrições e seguir melhorando a vigilância epidemiológica. Devem ser feitos preparativos de emergência e mais recursos materiais devem ser adicionados para dar suporte ao sistema de saúde. Esta previsão é provisória e depende das variáveis intervenientes se manterem constantes e qualquer alteração modificará sua previsão.

## Abstract (es)

**Objetivo:**

explorar el mejor tipo de curva o modelo de tendencia que podría explicar el comportamiento epidemiológico del contagio por COVID-19 y derivar las posibles causas que contribuyan a explicar el modelo correspondiente y las implicaciones sanitarias que se pueden inferir.

**Método:**

los datos fueron recogidos desde los informes COVID-19 del Departamento de Epidemiología, Ministerio de Salud, Chile. Los datos fueron sometidos a estudios de ajustes de curva en cuatro modelos diferentes: cuadrático, exponencial, exponencial suavizado simple y exponencial suavizado doble. El nivel de significación empleado fue de α≤0,05.

**Resultados:**

la curva que más se ajusta a la evolución de los casos confirmados acumulados del COVID-19 en Chile es la curva doble exponencial suave.

**Conclusión:**

el número de contagiados seguirá en aumento. Chile debe permanecer atento y ajustar las estrategias en torno a las medidas de prevención y control. El comportamiento poblacional juega un rol fundamental. Sugerimos no relajar las restricciones y seguir mejorando la vigilancia epidemiológica. Se deben hacer preparativos de emergencia y sumar más elementos de resorte al actual soporte sanitario. Esta predicción es tentativa y depende de que se mantengan todas las variables intervinientes constantes. Cualquier alteración modificará la predicción.

## Introduction

The rising pneumonia called COVID-19, caused by SARS-CoV-2, exhibits strong infectivity but less virulence when compared to SARS-CoV-1 and MERS-CoV in terms of morbidity and mortality. It is not only a virus that spreads from one person to another, but probably spreads because many people become infected in various places through different mechanisms. Restricting the movement of people, reducing contact, disseminating key high-frequency prevention information through multiple channels, mobilizing state and local authorities to respond quickly to the contingency, can help contain the pandemic^(1-5)^.

The actual number of infected cases is much larger than that reported worldwide. The observed mortality rate of COVID-19 is estimated at around 4.8% worldwide. Although this rate is low in Chile, this estimate may be incorrect due to underestimation, because the likelihood that the health authorities will collect severe cases is higher and, as active cases increase, the health resources do not support the demand and overestimation, considering that the vast majority of cases without symptoms or with mild symptoms are not investigated^(6-8)^.

The COVID-19 pandemic is an important international test for the medical and scientific community, as it reveals weaknesses in the management of emerging viral diseases and reminds us that contagious diseases should never be underestimated. In addition, it has strained health systems due to the virulence and excess demand on hospitals^(9-10)^.

It is essential to understand the transmission dynamics of the infection, as it could determine whether outbreak control measures are exerting a significant effect. The numbers of newly infected cases largely depend on the effectiveness of the control measures. Several governments have rapidly incorporated recent scientific findings into public policies at community, regional and national levels to slow down and/or prevent the further spread of COVID-19. Control measures such as quarantine, travel restrictions and airport inspections for travellers have been widely implemented to contain the spread of infections. The effectiveness of these containment measures to control the outbreak is inconclusive though^(1,3,5-6,11)^.

It is advisable for government entities to report on the current state of the pandemic in daily reports, specifying hospitalized and critical patients with COVID-19. Statistics need to be read carefully though, as it is leading to massive panic without organized solutions related to the redistribution of resources^(12)^.

Understanding the epidemiological characteristics of COVID-19 transmission in Chile is essential to formulate effective control strategies. The objective is to know what type of curve or model can best explain the epidemiological behavior of the accumulated confirmed cases of COVID-19 and identify the possible causes that contribute to explain the corresponding model and the health implications that can be inferred.

## Method

Data were collected from the COVID-19 reports from the Department of Epidemiology of the Ministry of Health in Chile (MINSAL). The data are publicly available^(7)^.

Curve adjustment studies were developed with the data in four different models: quadratic^(13)^, exponential^(13-14)^, simple exponential smoothing using the formula $[F_{t}=\;F_{\left(t-1\right)}\;+\;\alpha \;–\;A_{\left(t-1\right)}]\;$ where F_t_ = new prognosis, F _(t-1)_ = earlier prognosis and A_(t-1)_ = actual value of the earlier prognosis and double exponential smoothing using Holt’s method with trend adjustment $[{\mathrm{FIT}}_{t}=\;F_{t}\;+\;T_{t}]\;$ where FIT_t_ is the forecasted value]; the components of this formula are: $[\mathrm{Ft}=F_{\left(t-1\right)}\;+\;\alpha \;(A_{\left(t-1\right)}\;-\;F_{\left(t-1\right)})]\;y\;[T_{t=}\;\delta \;(F_{t-}\;F_{\left(t-1\right)})\;+\;(1-\delta )\;{T_{t}}^{\left(15-17\right)}$ .The following were estimated: the mean absolute percentage error (MAPE); the mean absolute deviation (MAD); the mean squared deviation (MSD). Criterion for choosing the best curve: small error coefficients. Indicator α is the weighting used in the level component of the smoothened estimate and δ is the weighting used in the trend component of the smoothened estimate (similar to a moving average of the differences between consecutive observations)^(15-16)^. To adjust the level of smoothing of the data (elimination of irregular fluctuations), the optimal ARIMA model was used for weighting, minimizing the sum of the square residues^(18-19)^. The absolute error of each measure was the difference (∆) between the actual observed value and the predicted value of confirmed cases for the same day. The median and interquartile range were estimated after checking for the normality of the absolute errors using the Kolmogorov-Smirnov test. Minitab 18.0^®^ software was used. The significance level was α ≤ 0.05.

## Results

**Figure 1:**
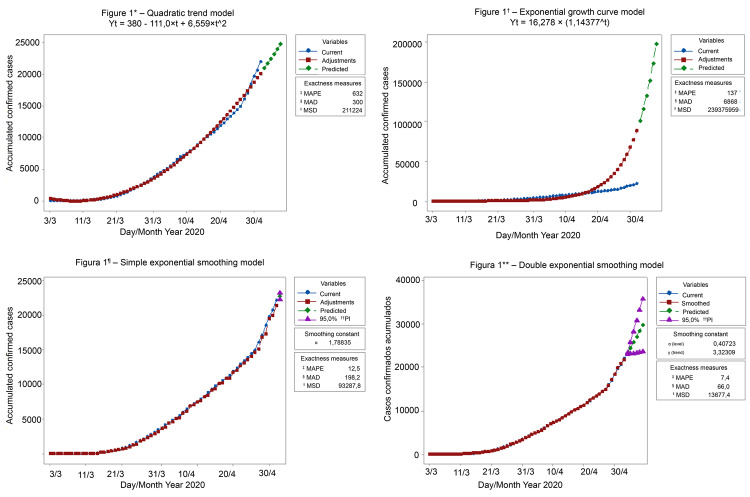
– Estimation results of the observed data curves of confirmed, adjusted and predicted cases, according to the model. Chile, 2020 Figure 1* = quadratic; Figure 1^†^ = exponential; ^‡^MAPE = mean absolute percentage error; ^**§**^MAD = mean absolute deviation; ^ǁ^MSD = mean squared deviation. Figure 1^**¶**^ = simple exponential smoothing; Figure 1** = double exponential smoothing; ^††^IP = prediction interval

Figure 1 presents the estimated results of the regression equations of the observed data curves of confirmed, adjusted and predicted cases in the quadratic, exponential, simple exponential smoothing and double exponential smoothing models. The MAD, MAPE, and MSD coefficients are lower in the double exponential smoothing curve, which shows that the curve that best fits the evolution of the accumulated confirmed cases of COVID-19 in Chile is the one described above.

Table 1 presents the results of estimating the predicted value on the previous day of confirmed cases (with its corresponding confidence interval) and the actual result of confirmed cases that occurred on the predicted day. The results of actual confirmed cases differ little from the predicted value (S-W = 0.907; median = 53.2 and interquartile range= 72.80) and, with some exceptions, the actual value was within the estimated confidence interval for the predicted day.

**Table 1:** – Estimation results of the predicted value on the previous day of confirmed cases (with their corresponding confidence interval) and the actual result of confirmed cases that occurred on the predicted day, using the double exponential smoothing method. Chile, 2020

Period	Prognosis [^*^95% CI]	(CC^†^)
03-23-2020	763.25	746
03-24-2020	883.53	922
25-03-2020	1053.34	1142
03-26-2020	1360.68 [1269.68 ; 1451.69]	1306
03-27-2020	1428.48 [1338.48 ; 1518.48]	1610
03-28-2020	1823.25 [1660.25 ; 1987.12]	1909
03-29-2020	2172.58 [1985.46 ; 2359.70]	2139
03-30-2020	2421.80 [2222.44 ; 2621.16]	2449
03-31-2020	2726.89 [2516.05 ; 2937.74]	2738
04-01-2020	3026.17 [2807.06 ; 3245.28]	3031
04-02-2020	3323.42 [3097.77 ; 3549.07]	3404
04-03-2020	3713.18 [3471.18 ; 3955.18]	3737
04-04-2020	4058.70 [3926.76 ; 4190.63]	4161
04-05-2020	4566.29 [4430.67 ; 4701.55]	4471
04-06-2020	4798.07 [4659.68 ; 4936.46]	4815
04-07-2020	5162.60 [5028.60 ; 5296.66]	5116
04-08-2020	5431.90 [5298.90 ; 5564.91]	5546
04-09-2020	5934.95 [5797.49 ; 6072.42]	5972
04-10-2020	6376.54 [6240.11 ; 6512.67]	6501
04-11-2020	6986.93 [6844.31 ; 7129.54]	6927
04-12-2020	7368.19 [7226.58 ; 7509.80]	7213
04-13-2020	7554.50 [7405.20 ; 7703.70]	7525
04-14-2020	7865.82 [7718.89 ; 8012.80]	7917
04-15-2020	8297.20 [8151.20 ; 8443.10]	8273
04-16-2020	8627.80 [8484.48 ; 8771.10]	8807
04-17-2020	9281.20 [9112.70 ; 9332.80]	9252
04-18-2020	9703.80 [9608.90 ; 9798.70]	9730
04-19-2020	10198.50 [10104.50 ; 10292.60]	10088
04-20-2020	10492.60 [10394.90 ; 10590.20]	10507
04-21-2020	10920.00 [10823.90 ; 11016.40]	10832
04-22-2020	11193.90 [11095.50 ; 11292.30]	11296
04-23-2020	11711.30 [11610.90 ; 11812.40]	11812
04-24-2020	12283.70 [12179.50 ; 12387.90]	12306
04-25-2020	12786.80 [12686.50 ; 12893.00]	12858
04-26-2020	13384.20 [13280.20 ; 13488.20]	13331
04-27-2020	13826.10 [13721.60 ; 13923.50]	13813
04-28-2020	14300.60 [14197.40 ; 14403.80]	14365
04-29-2020	14888.50 [14784.40 ; 14992.70]	14885
04-30-2020	15406.40 [15303.90 ; 15508.90]	16023
05-01-2020	17006.90 [16873.40 ; 17140.50]	17008
05-02-2020	18356.20 [18224.90 ; 18487.60]	18435
05-03-2020	19885.00 [19720.50 ; 19989.50]	19663
05-04-2020	20976.10 [20837.80 ; 21114.40]	20643
05-05-2020	21554.60 [21404.30 ; 21704.90]	22016

*CI = Confidence interval; ^†^CC = Confirmed cases

**Figure 2:**
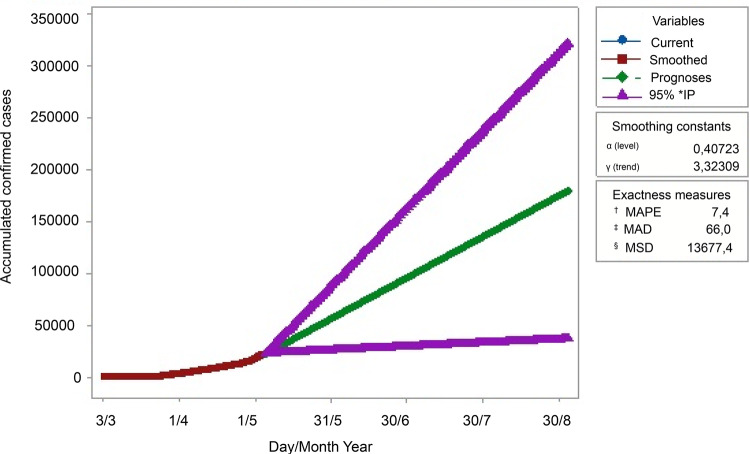
– Estimation results of confirmed cases from the present until August 30th, 2020 (Forecast). Chile, 2020 * IP = prediction interval; ^†^MAPE = Mean absolute percentage error; ^**‡**^MAD = Mean absolute deviation; ^**§**^MSD = Mean squared deviation

Figure 2 and Table 2 present the estimation results of the forecasted number of confirmed cases from March 3^rd^, 2020 to August 30^th^, 2020. The MAPE coefficients are the lowest in the double exponential smoothing curve and the same is true for the MAD and MSD coefficients, which are low and acceptable. As observed, the confidence intervals increase as predicted further ahead and, therefore, the estimation error increases^(18)^.

**Table 2:** – Estimation results of confirmed cases from the present to August 30th, 2020, selected dates (forecasts) using the double exponential smoothing model. Chile, 2020

Period	Forecast	LL* - CI^†^	UL^‡^ - CI^†^
05-06-2020	23083	22921	23245
05-07-2020	24391	23074	25708
05-08-2020	25699	23193	28204
05-09-2020	27006	23310	30703
05-10-2020	28314	23427	33201
05-11-2020	29622	23544	35700
05-12-2020	30930	23661	38199
05-13-2020	32238	23778	40698
05-14-2020	33546	23895	43197
05-15-2020	34854	24012	45696
05-16-2020	36162	24129	48195
05-17-2020	37469	24245	50693
05-18-2020	38777	24362	53192
05-19-2020	40085	24479	55691
05-20-2020	41393	24596	58190
05-21-2020	42701	24713	60689
05-22-2020	44009	24829	63188
05-23-2020	45317	24946	65687
05-24-2020	46625	25063	68186
05-25-2020	47932	25180	70685
05-26-2020	49240	25296	73184
05-27-2020	50548	25413	75683
05-28-2020	51856	25530	78182
05-29-2020	53164	25647	80681
05-30-2020	54472	25763	83180
06-10-2020	68858	27048	110669
06-20-2020	81937	28215	135659
06-30-2020	95016	29383	160649
07-10-2020	108094	30551	185638
07-20-2020	121173	31718	210628
07-30-2020	134252	32886	235618
08-10-2020	148638	34170	263107
08-20-2020	161717	35337	288097
08-30-2020	174796	36505	313087

*LL: Lower limit; ^†^ CI: Confidence interval; ^‡^ UL: Upper limit

## Discussion

The progress of the COVID-19 pandemic in Chile fits well into a model and we study its predictive capacity. By analyzing the epidemiological characteristics and transmission dynamics of an emerging infectious disease, the key to successful outbreak control is obtained through mitigation strategies^(6)^.

The World Health Organization (WHO) has indicated that easing restrictions does not end the epidemic in any country. Ending the epidemic will require a sustained effort by individuals, communities and governments to continue to fight and control the virus. Active surveillance is recommended to detect cases early and isolate them, quickly locate those who have been in contact with the cases and monitor them so that patients can quickly access clinical care. If none of these points are achieved, any action or indication will be ineffective in containing the pandemic. Chile has disseminated neither the notified contact indicators nor the indicator of test results delivered within 24 hours. Contacts are being notified, but without any specification of the time. There are no statistics either with regard to compliance with quarantines or supervised discharge of confirmed patients^(1,7,20-21)^.

WHO has provided support for epidemiological studies of seroprevalence, which suggest that the percentage of the population infected with antibodies may be relatively small (2-3%), without knowing how long this immunity lasts^(20)^.

The mathematical model of any process tries to describe its basic components and tries to predict some general trends, but it will never be able to provide an exact description and prediction^(22)^. Various causes impede this in an epidemiological context: (a) no model can include all variables that influence a process; (b) there are unknown variables that cannot be incorporated in the prediction; (c) these variables depend on conditions and the specific nature of the virus, on the social conditions people live in, and on the immunity status of a particular patient; (d) the socio-economic infrastructure of a country; (e) the capacities of the health system at a given time, f) a smart health policy that a country could implement to prevent the spread and take the most appropriate measures to mitigate the impact of the process associated to these infections to the maximum; and (g) on the intelligence and level of scientific knowledge of the authorities at all levels; (h) the authorities’ ability to learn from experiences observed in other parts of the world. The knowledge of these variables and the consequent intentional modification of all or some of these variables will modify the contagion rate in a positive or negative way. Therefore, this rate and the specific derivations of the nature of all of these variables make it impossible to create an exact forecast of the future. Thus, the model presented here can only provide a basis for a comprehension mechanism, under the type of circumstances, constraints and current population conditions. All of these aspects limit the predictive capacity and attention needs to be paid to changes in the determining factors that may constitute the cause compelling to change the model to explain the behavior of the pandemic. One of the transcendental aspects in epidemiology is to try to predict the evolution of infectious diseases. This attempt is usually done through models that consider the progress of cases over time in a certain place, transmissibility, among other causes. Nevertheless, these do not include the specific characteristics of the affected population^(23-25)^.

The experience of European countries that have indicated specific population intervention measures has shown that the deceleration of the incidence is achieved when a percentage or critical mass of confirmed cases is reached, compared to the general population. That is the case for: Austria (0.12%), Norway (0.12%), Netherlands (0.13%), Germany (0.14%), France (0.15%), Denmark (0.15%), Switzerland (0.17%), United Kingdom (0.18%), Portugal (0.18%), Iceland (0.18%), Sweden (0.20%), Spain (0.21%) and Italy (0.21%). It is estimated that the slowdown in the incidence in Chile would be achieved when the average of 31.527 (SD=12.160) confirmed cases is reached. That happens on May 13^th^, 2020 (May 10^th^ and July 19^th^). It should be mentioned that active cases in Chile represent 47.04% (SD=1.96) of confirmed cases, as of May 5^th^, 2020. The propensity of this percentage should tend to decrease gradually and slowly according to international experience, but it has stagnated for 13 days between 45-50%. This stagnation is probably due to the fact that the public health strategies and the population’s behavior are not equivalent to that of the countries mentioned^(26-38)^.

Chile needs to remain vigilant and adjust strategies around prevention and control measures, which means to improve health actions and to strengthen surveillance systems and public health infrastructure to provide the early detection and rapid response. We hope, for the sake of Chilean health, that the support of infrastructure and health assets achieve the results in terms of effectiveness and do not pose a problem of needs and resources. At the same time, emergency preparations are necessary in response to a more serious outbreak that may occur^(1,6)^.

The monitoring of simple but at the same time critical parameters of patient behavior at an intensive care unit (ICU) grants us critical information to know these parameters and their evolution in terms of severity, providing information for the sake of control, intervention and mitigation strategy proposals. The current use of the intensive care capacity for COVID-19 patients represents on average 17.10% (SD=1.35) of the total available ICU beds in Chile. Whether the country reaches 20,111 active cases (42,751 confirmed cases, according to the active case/confirmed case ratio), the number of intensive care beds will not be sufficient for COVID-19 patients and this health support would collapse. This would happen on May 22^nd^, 2020. This calculation considers the current number of intensive care beds available in Chile: 3,264 and the average percentage of intensive care capacity use for COVID-19 patients in Chile, which is 8.28% (SD=1.06), and intensive care capacity use in non-COVID-19 patients, which is 48.96% (SD=1.74)^(39-41)^. If the number of ICU beds remains constant and the incidence does not decelerate (as it falls within the error margin of the prediction), but the ratio of active cases/confirmed cases decreases, then the scenario of ICU collapse is likely.

Ventilation support is the cornerstone of the management of subjects with respiratory failure due to COVID-19. The current use of invasive mechanical ventilation (IMV) for COVID-19 patients represents on average 18.48% (SD=1.22) of the total mechanical ventilators (MV) available in Chile. If the country reaches 28,423 active cases (60,418 confirmed cases, according to the active case/confirmed case ratio), MV will not be sufficient for the ventilatory needs of COVID-19 patients and this health support would collapse. This would happen on June 4^th^, 2020. This calculation considers the current number of MV available in Chile: 1,825 and the average percentages in Chile of IMV use in COVID-19 patients, which is 5.01% (SD=0.67), and of IMV use in non-COVID-19 patients, which is 21.91% (SD=1.17) ^(41-42)^. If the number of MV remains constant and the incidence rate (as it falls within the error margin of the prediction) and the active case/confirmed case ratio do not decrease, the scenario of health failure due to the lack of MV is plausible, although less likely than the collapse of the intensive care system.

This predictive model is provisional and depends on the constant use of all the variables maintained. Any change will undoubtedly modify the prediction and, therefore, daily variations should be monitored, although the predictive model does not present considerable and important faults in the current reality. The predictions of this model have been good and have a safety coefficient that allows us to calculate with some slack the public health needs the care for the current cases might require. This information is relevant when deciding on measures to contain an epidemic. The appropriate fit and good predictive capacity of this model makes it possible to propose it as an epidemiological method for monitoring and predicting the progression of infectious diseases in the local situation, e.g. regions, provinces and communes^(43)^.

Our approach has limitations. This study was based on the cases reported by MINSAL and contains the biases of case confirmation and information, which may be delayed with respect to the occurrence of cases, the delay in the appearance of symptoms due to the incubation period and the high proportion of unreported cases as a result of limited detection and testing capacity. Moreover, data sources may be biased, incomplete or only capture certain aspects, versus others that may be equally relevant, such as chronic pathologies, risk factors, nutritional status, among others.

This analysis does not necessarily represent the actual situation of the cases, as it excludes confirmed cases in laboratories not considered by surveillance systems and sub-clinical cases. This model does not consider variations due to seasonal changes, social aggregation, etc. either. In addition, it is dependent on the addition of new MV to the health network. This analysis of epidemiological characteristics provides important information on the behavior of COVID-19 to propose effective control strategies at all levels of health care^(7)^.

## Conclusion

The type of curve that best explains the behavior of COVID-19 in Chile is a doubly smoothed exponential curve. From this model, it follows that the number of infected cases will continue to increase. The number of confirmed cases will grow stronger and within a short time if the population behavior and public health measures are not in line with the size of this international public health emergency. We hope that the results will enable the medical staff and leaders to make decisions. In no case should restrictions be relaxed and epidemiological surveillance needs improvements, as this is not the end of the pandemic.
